# Cost-Utility Analysis of Trastuzumab-Emtansine Versus Trastuzumab for the Treatment of Residual Invasive HER-2-Positive Breast Cancer in Iran

**DOI:** 10.5812/ijpr-153452

**Published:** 2024-12-17

**Authors:** Homa Hemati, Marzieh Nosrati, Meysam Seyedifar

**Affiliations:** 1 Department of Pharmacoeconomics and Pharmaceutical Administration, Faculty of Pharmacy, Tehran University of Medical Sciences, Tehran, Iran; 2 Personalized Medicine Research Center, Endocrinology and Metabolism Clinical Sciences Institute, Tehran University of Medical Sciences, Tehran, Iran; 3 Pharmaceutical Management and Economic Research Center, The Institute of Pharmaceutical Sciences (TIPS), Tehran University of Medical Sciences, Tehran, Iran

**Keywords:** HER-2-Positive Breast Cancer, Cost-Utility Analysis, Budget Impact Analysis, Breast Cancer, Economic Evaluation, Trastuzumab Emtansine

## Abstract

**Background:**

Breast cancer is one of the most common types of cancer in women, and its incidence is increasing in Iran. HER-2-positive breast cancer is invasive and often associated with poorer outcomes. Patients with this type of breast cancer can develop resistance to medications like trastuzumab. Trastuzumab-emtansine (TDM1) is a medication developed to reduce cancer cell resistance to trastuzumab. The TDM1 has been shown to decrease the incidence of death and recurrence in breast cancer.

**Objectives:**

This study aimed to evaluate the cost-utility and calculate the budget impact of TDM1 versus trastuzumab for the treatment of residual invasive HER-2-positive breast cancer.

**Methods:**

A Markov model with a lifetime horizon was developed, incorporating four health states. Women aged 45 with residual invasive HER-2-positive breast cancer entered the model. The study adopted a healthcare system perspective, with costs reported in 2021 US dollars. Discount rates of 7% for costs and 3% for utility values were applied. Utility values and transition probabilities were derived from published literature. Costs were estimated based on guidelines, expert opinions, and Iranian tariffs. Iran’s pharmacoeconomic threshold of 1085$ was used for comparison. The incremental cost-effectiveness ratio (ICER) and budget impact of TDM1 were calculated, and sensitivity analyses were conducted to assess the robustness of the model.

**Results:**

The model indicated that treatment with TDM1 resulted in a 1.59 quality-adjusted life year (QALY) increase, with an additional cost of 1408$. This was deemed cost-effective, considering Iran’s pharmacoeconomic threshold of 1085$ (calculated ICER: 886$ per QALY gained). One-way sensitivity analysis revealed that the model was sensitive to the costs of TDM1 and trastuzumab, the discount rates for utility values and costs, and the probability of achieving invasive disease-free survival (IDFS). Probabilistic sensitivity analysis showed that 59.61% of simulations fell below Iran’s pharmacoeconomic threshold, supporting the model's robustness. The budget impact analysis revealed that the additional budget required for TDM1 treatment over a three-year period was 1,120,546$ compared to trastuzumab.

**Conclusions:**

Although TDM1 imposes higher costs, it is more cost-effective than trastuzumab for the treatment of residual invasive HER-2-positive breast cancer in Iran.

## 1. Background

Breast cancer is the most common type of cancer worldwide (1), with 2.3 million individuals diagnosed annually (2). Moreover, the incidence and mortality rates of breast cancer have risen over the past three decades. Deaths due to breast cancer are more frequently reported in developing countries, with an incidence rate approximately 88% higher than in developed nations. If current mortality trends persist, low- and middle-income countries are projected to account for 75% of all global breast cancer deaths by 2030 (3, 4). Similar to other developing countries, the incidence of breast cancer is increasing in Iran, where it is one of the most common cancers among women (5). 

Breast cancer places a significant economic burden on societies. The overall ten-year living costs for a breast cancer patient exceed those for liver, cervical, colorectal, and lung cancer (6). In Iran, treatment costs for breast cancer vary depending on the disease stage, averaging approximately 222.17$, 224.61$, 316.51$, and 828.52$ per patient for stages I through IV, respectively (7). A study assessing breast cancer-related direct and indirect costs in Iran estimated the economic burden of the disease to be around 947,374,468$. Approximately 77% of these costs were attributed to reduced productivity among patients, while about 18.5% were related to direct costs. Among all direct costs, chemotherapy drugs accounted for the largest share, amounting to 76,755,740$ (8).

This disease is heterogeneous and can be classified into clinical subgroups based on its molecular features. Approximately 15 to 20 percent of breast cancers belong to the HER-2 positive subgroup (9, 10). This subtype is invasive and often associated with poorer treatment outcomes and drug resistance. Although incorporating HER-2-targeted therapies into treatment regimens has improved clinical results, resistance to these therapies remains a challenge (10).

Trastuzumab, in combination with other standard therapeutic drugs, is commonly used to treat HER-2 positive breast cancer (1). However, resistance to trastuzumab is prevalent, with many patients experiencing disease recurrence within a year. To address this issue, alternative treatments such as trastuzumab-emtansine (TDM1) have been developed to reduce cancer cell resistance to trastuzumab (11). Emtansine, or DM1, is an antimicrobial agent derived from the Maitansine molecule. Trastuzumab-emtansine, marketed under the brand name Kadcyla, is an antibody-drug conjugate that facilitates drug delivery to HER-2 positive cells, effectively reducing their resistance to trastuzumab (10).

Trastuzumab-emtansine has demonstrated its ability to lower the incidence of breast cancer-related deaths and recurrences (12). However, TDM1 is more expensive than trastuzumab, and its cost-effectiveness evaluations have yielded varied results globally (13, 14). Consequently, it is crucial to assess its cost-effectiveness within the Iranian context before considering its inclusion in Iran's drug list.

## 2. Objectives

The objective of this research was to evaluate the cost-utility and budget impact of TDM1 compared to trastuzumab for treating patients with residual invasive HER-2 positive breast cancer.

## 3. Methods

### 3.1. Decision Model

**Figure 1.**
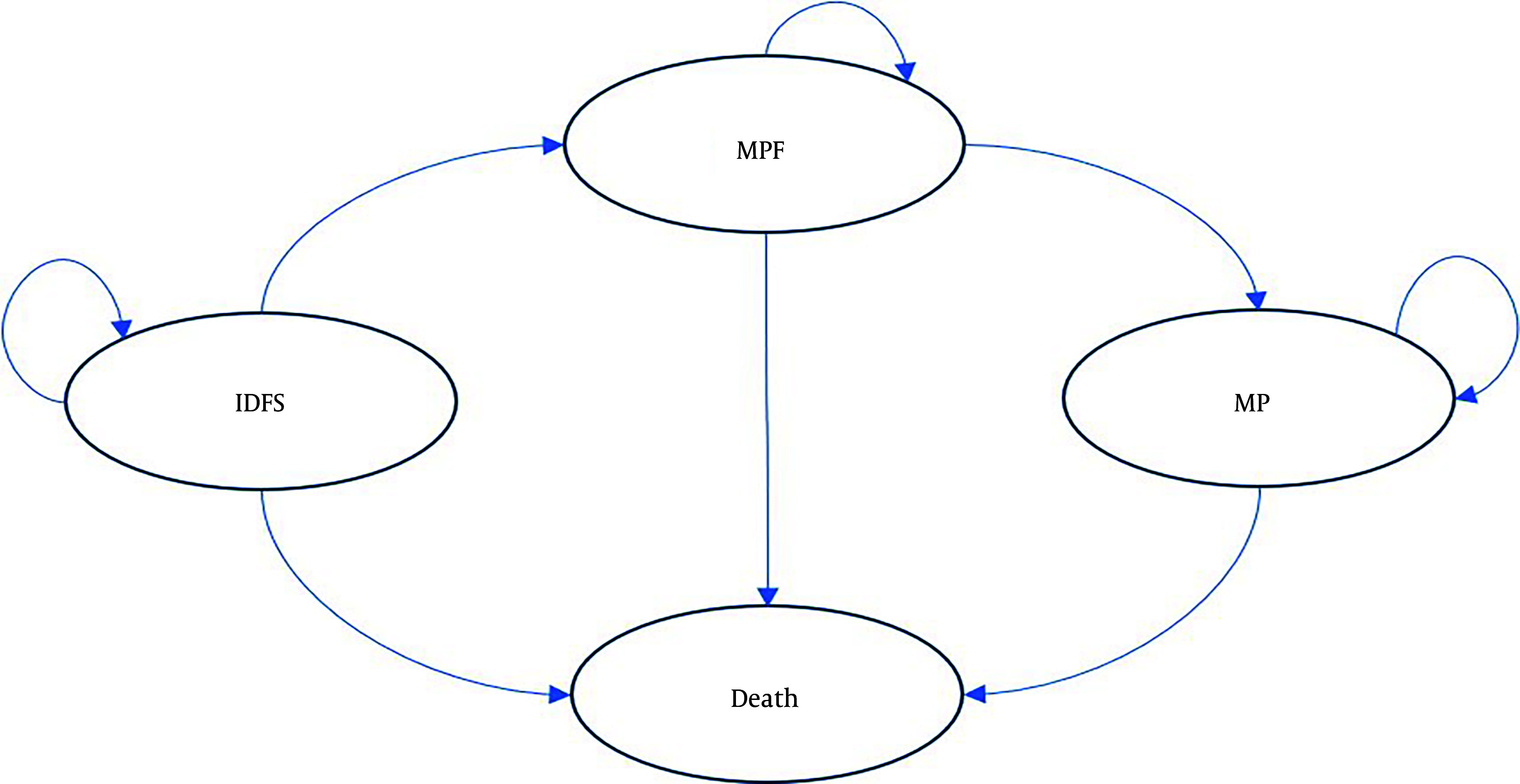
Markov model for patients with residual invasive HER-2+ breast cancer. Invasive disease-free survival (IDFS), metastatic progression-free (MPF), metastatic progression (MP)

In this study, we performed a cost-utility analysis and developed a Markov model in Excel 2021 to compare the costs and outcomes of T-DM1 versus trastuzumab in the treatment of invasive breast cancer with HER-2-positive cells (Figure 1). We adopted a lifetime horizon with a discount rate of 7% for costs and 3% for utilities (15, 16). This study utilized a healthcare system perspective. All costs were converted from Iranian Rials (IRR) to US dollars based on the exchange rate at the time of the study (1 IRR = 0.0000045 USD) (17). For the base-case analysis, a hypothetical cohort of 1,000 45-year-old women with residual invasive HER-2-positive breast cancer who had received neoadjuvant treatment was modeled.

The Markov model consisted of four health states: Invasive disease-free survival (IDFS), metastatic progression-free (MPF), metastatic progression (MP), and Death. The cycle length of the Markov model was set at 21 days. In this model, patients started treatment in the IDFS state. They could remain in this state, enter the MPF state, or die from causes unrelated to breast cancer. Patients in the MPF state could either remain in this state, enter the MP state, or die. Similarly, patients in the MP state could either remain in this state or die.

### 3.2. Outcomes

The outcomes of interest in this study included IDFS, progression-free survival (PFS), distant recurrence-free survival, overall survival (OS), the safety profile of TDM1 compared to trastuzumab, costs (reported in 2021 US dollars), quality-adjusted life years (QALYs), and the incremental cost per QALY. A systematic review was conducted to identify relevant clinical data for the analysis. The clinical data incorporated into the model were extracted from published literature identified during this review.

### 3.3. Probabilities

Using the Kaplan–Meier curve from previously published studies, the probabilities of primary metastasis occurrence, progression to the MPF state, the MP state, and other relevant probabilities for the TDM1 and trastuzumab arms were extracted (18-20). The probability of natural death in women aged 45 and above was calculated using data from the WHO life table. Probabilities of various side effects were obtained from published literature. Table 1 presents the probabilities used in the model.

**Table 1.** Model Parameters

Parameters, (References)	Base Case Value (95% Confidence Interval)	Distribution
**Daily dose**		
**Trastuzumab, (** 18 **)**	6 mg/kg every 3 weeks for 14 cycles	Normal
**TDM1, (** 18 **)**	3.6 mg/kg every 3 weeks for 14 cycles	Normal
**Docetaxel, (** 19 **)**	75 or 100 mg/m^2^ every 3 weeks for a minimum of 6 cycles	Normal
**Probabilities (annual)**		Beta
IDFS (hazard ratio), (18)	0.5 (0.39 - 0.64)	
Distant recurrence free survival, (18)	0.6 (0.45 - 0.79)	
OS (MPF state), (19)	0.74 (0.49 - 1.120)	
PFS (MPF state), (19)	0.69 (0.48 - 0.990)	
**Trastuzumab side effects % (> grade III), (** 18 **)**		Beta
Decreased platelet count	0.3	
Hypertension	1.2	
Radiation-related skin injury	1	
Peripheral sensory neuropathy	D	
Decreased neutrophil count	0.7	
Hypokalemia	0.1	
Fatigue	0.1	
Anemia	0.1	
**TDM1 side effects % (> grade III), (** 18 **)**		Beta
Decreased platelet count	5.7	
Hypertension	2	
Radiation-related skin injury	1.4	
Peripheral sensory neuropathy	1.4	
Decreased neutrophil count	1.2	
Hypokalemia	1.2	
Fatigue	1.1	
Anemia	1.1	
**Trastuzumab + docetaxel side effects % (> grade III), (19)**		Beta
Diarrhea	4.2	
Peripheral neuropathy	28	
Peripheral edema	27.8	
Neutropenia	19.3	
Febrile neutropenia	6.5	
Elevated ALT	0.8	
Elevated AST	0.3	
Elevated GGT	0.3	
Anemia	5	
Hypertension	4.7	
**Utilities**		Beta
IDFS off-treatment, (21)	0.826	
IDFS on-treatment, (21)	0.814	
MPF, (22)	0.702	
MP, (22)	0.443	
EOL, (23)	0.250	
**Adverse events **		Beta
Neutropenia, (24)	-0.09	
Fatigue, (22)	-0.115	
Anemia, (25)	-0.12	
platelet count decreased, (26)	-0.108	
Peripheral neuropathy, (27)	-0.12	
**Trastuzumab + docetaxel side effects**		Beta
Diarrhea, (22)	-0.103	
Peripheral neuropathy, (27)	-0.12	
Peripheral edema, (27)	-0.06	
Neutropenia, (24)	-0.09	
Febrile neutropenia, (22)	-0.15	
Anemia, (28)	-0.12	
**Costs, US dollars**		
Cost of medicines (calculated in this study)		Gamma
Trastuzumab (150 mg vial)	71$	
Trastuzumab (440 mg vial)	182$	
TDM1 (one vial)	180$	
Docetaxel (20 mg vial)	8$	
Docetaxel (80 mg vial)	31$	
Cost of monitoring, (calculated in this study)		Gamma
Trastuzumab/TDM1	13$	
Docetaxel	6$	

Abbreviations: ALT, alanine transaminase; AST, aspartate aminotransferase; EOL, end of life; GGT, gamma-glutamyl transferase; IDFS, invasive disease-free survival; MPF, metastatic progression-free; MP, metastatic progression; OS, overall survival; PFS, progression-free survival; TDM1, trastuzumab-emtansine.

### 3.4. Health State Utilities

In the IDFS state, patients are either receiving medical treatment (trastuzumab or TDM1) or not receiving medical treatment. Patients in the on-treatment state have different utility values compared to those in the off-treatment state due to the side effects of the drugs. The base utility values for the on-treatment and off-treatment states are 0.814 and 0.826, respectively (21). Utility values for other health states were calculated by accounting for the disutilities associated with medication side effects. The utility values for metastatic breast cancer patients in the MP, MPF, and EOL states were obtained from published literature (22, 23). The overall utility values for each state are shown in Table 1. 

### 3.5. Costs

Costs were calculated from a healthcare system perspective and expressed in 2021 US dollars. This study included the direct medical costs, encompassing the costs of medication, monitoring, side effects prevention, side effects management, and physician visits. 

In Iran’s pharmaceutical market, both generic and brand-name medicines are available. Therefore, the calculation of medical costs was conducted by considering the market shares of generic and brand-name medicines, as reported in Iran's official pharmaceutical statistics. The market share for trastuzumab is 95% for the generic version and 5% for the brand-name version. The average weight of a 55-year-old Iranian woman (65 kg) was used to calculate the medication dosage (29). After consulting with experts, the monitoring costs for trastuzumab, TDM1, and docetaxel were determined. 

Management of side effects rated above grade III was factored into the cost calculations for each treatment arm. According to published literature and consultations with healthcare professionals, when side effects above grade III occur, the medication dose is either reduced or discontinued. The only side effect incurring additional costs in this model is neutropenic fever (30). Dose reductions for trastuzumab and TDM1 due to side effects were based on the Katherine trial (18). The cost of antibiotic treatment for neutropenic fever was included in the model. 

For calculating direct medical costs, 80% of government tariffs and 20% of private tariffs were applied.

Following the occurrence of metastasis, patients undergo a docetaxel-trastuzumab regimen. The dosing and administration of these two medicines were based on a study involving patients with metastatic HER-2-positive breast cancer (19). The cost of medication preparation and injection per session is estimated at 4.5$, as determined through consultation with healthcare professionals. Monitoring costs associated with docetaxel were included in the calculations. Additionally, dose reductions of docetaxel due to side effects were incorporated based on data from published literature (19).

For the MP health state, since the calculated costs of the two previous health states (IDFS and MPF) were approximately one-third of those reported in the study by Perez et al., we assumed that the cost of the MP health state in Iran is similarly one-third of the value calculated in the mentioned study (19). 

End-of-life (EOL) care costs for patients with metastatic breast cancer typically escalate during the last six months of life. The overall cost of care during this period is approximately 4.15 times higher than for patients in earlier health states prior to becoming metastatic. This increase is primarily attributable to cancer-related inpatient and hospice costs (31). Accordingly, we applied the coefficient of 4.15 to calculate the EOL care costs for this period.

### 3.6. Sensitivity Analysis

To assess the robustness of the model, a deterministic sensitivity analysis was conducted for the model inputs. Additionally, a probabilistic sensitivity analysis was performed in MS Excel 2022 using 10,000 simulations.

### 3.7. Budget Impact Analysis

A budget impact analysis was conducted to estimate the total financial implications of using TDM1 for the treatment of residual invasive HER-2-positive breast cancer over a three-year period. It was assumed that patients had only two treatment options. The budget impact was calculated from a healthcare system perspective, considering the direct medical costs derived from the cost-effectiveness model over 1-, 2-, and 3-year time horizons. The difference in cost per patient was multiplied by the annual number of eligible cancer patients to receive TDM1 in Iran. 

The prevalence of breast cancer, the number of treatment recipients, the proportion of HER-2-positive patients, and those eligible for either TDM1 or trastuzumab were estimated using published studies and expert opinions. The TDM1 market share was determined based on consultations with the marketing team of the manufacturer and healthcare professionals. Iranian experts estimated a total of 12,684 patients with breast cancer in Iran, with 95% of these patients having access to medical treatment. Among these, 24.3% were identified as HER-2-positive (32). Of the HER-2-positive breast cancer patients who received basic chemotherapy regimens, 40% to 60% (equivalent to 1,464 patients) were found to have residual malignant tissues requiring treatment with trastuzumab or TDM1 (33). 

An annual growth rate of 3% was applied for HER-2-positive and aggressive breast cancer cases, resulting in an estimated patient population of 1,508 in the second year and 1,553 in the third year. Based on consultations with marketing and sales managers from the importing company, the market share of TDM1 was assumed to be 5%, 10%, and 15% for the first three years, respectively, compared to trastuzumab. To simplify the model, it was assumed that all other patients were treated with trastuzumab, and only the costs associated with trastuzumab were considered for patients not receiving TDM1.

## 4. Results

### 4.1. Base Case Analysis

Table 2 summarizes the base-case analysis results. The total costs associated with the trastuzumab and TDM1 treatment arms were 9,968$ and 11,376$, respectively. The QALYs calculated for patients in the trastuzumab and TDM1 arms were 3.99 and 5.58, respectively. The incremental cost-effectiveness ratio (ICER) was calculated as 886$ per QALY gained.

Since TDM1 provides an improvement in QALY at a higher cost, the ICER must be evaluated against Iran's pharmacoeconomic threshold. At the time of the study, the pharmacoeconomic threshold in Iran was 1,085$ per QALY. As the calculated ICER is below this threshold, the administration of TDM1 is considered cost-effective in the Iranian healthcare setting.

**Table 2.** Base Case Analysis Results

Variables	Utility (QALY)	Cost ($)
**Trastuzumab**	3.99	9968
**TDM1**	5.58	11376
**ICER**	886$ per QALY	

Abbreviations: TDM1, trastuzumab-emtansine; ICER, incremental cost-effectiveness ratio; QALY, quality-adjusted life-year.

### 4.2. One-Way Sensitivity Analysis

**Figure 2.**
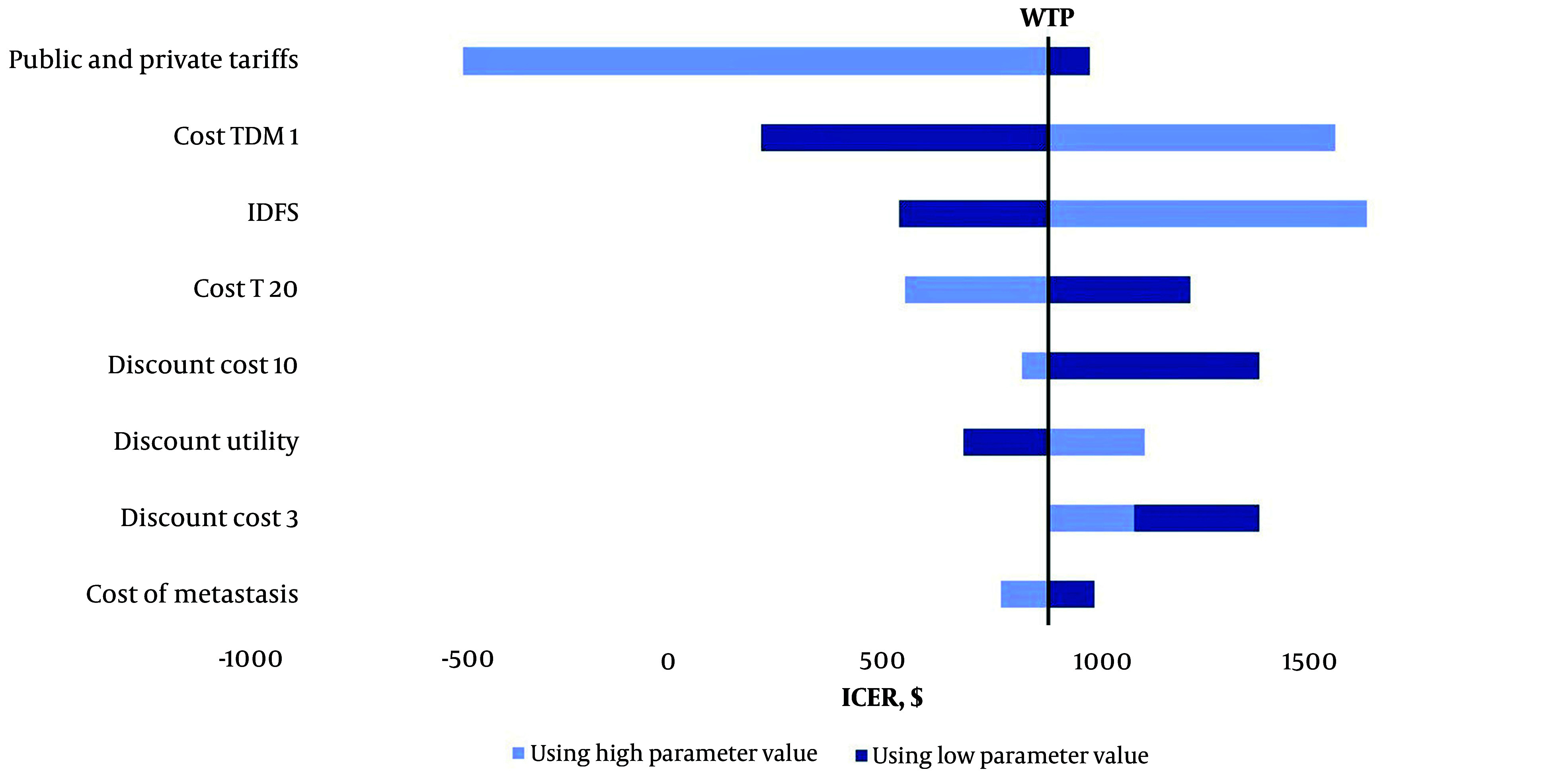
Tornado diagram. Incremental cost-effectiveness ratio (ICER), willingness to pay (WTP)

We conducted one-way deterministic sensitivity analyses to evaluate the impact of each parameter included in the model. Figure 2 presents a tornado diagram illustrating the results of these sensitivity analyses. When the cost of TDM1 was varied by ± 20%, the ICER ranged from 215$ to 1,557$. Similarly, varying the cost of trastuzumab by ± 20% resulted in ICER values between 552$ and 1,220$. Additionally, applying a ± 20% change in metastatic state costs led to ICER variations ranging from 777$ to 995$.

Regarding the discount rate for utilities, increasing the discount rate to 6% raised the ICER to 1,111$. When costs were discounted at 0%, 3%, and 10%, the ICER values were 1,380$, 1,088$, and 826$, respectively. Furthermore, adjusting the lower and upper limits of IDFS probabilities resulted in ICER values ranging from 537$ to 1,623$. 

Lastly, costs were recalculated without considering private tariffs, and this adjustment did not influence the overall results of the model.

### 4.3. Probabilistic Sensitivity Analysis

**Figure 3.**
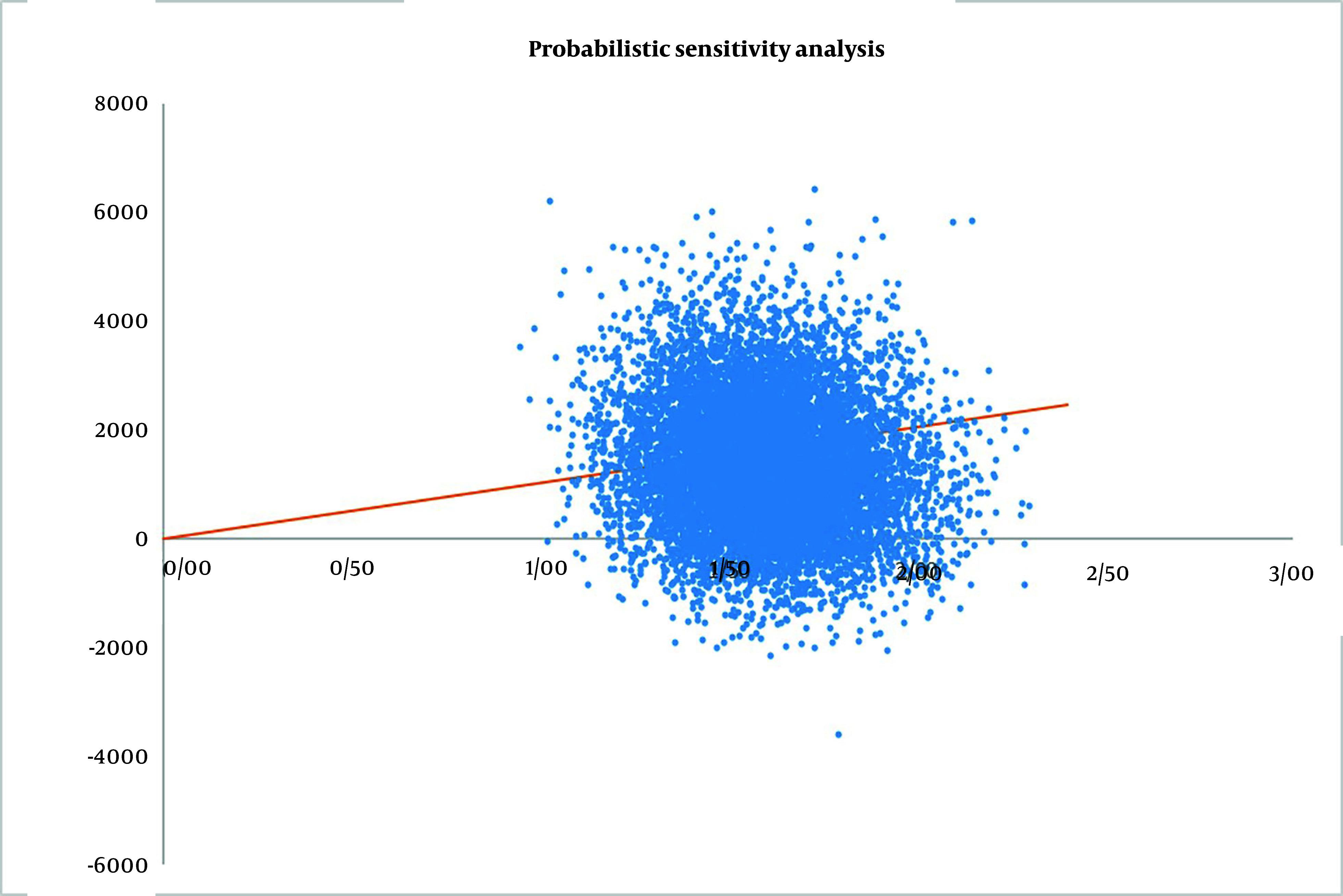
Probabilistic sensitivity analysis. The orange line is the willingness to pay threshold.

To assess the uncertainty of all model inputs, we conducted a probabilistic sensitivity analysis using Monte Carlo simulation. Figure 3 displays the distribution of ICER from 10,000 model iterations performed in Excel 2022. A total of 88.66% of simulations fall within the upper right-hand quadrant of the cost-effectiveness plane, with 59.61% of simulations below Iran’s pharmacoeconomic threshold, indicating that prescribing TDM1 is cost-effective in the majority of iterations. Additionally, 11.34% of the iterations fall within the lower right-hand quadrant of the cost-effectiveness plane, suggesting that TDM1 could be cost-saving while also being more effective than trastuzumab.

### 4.4. Budget Impact Outcomes

The results of the budget impact analysis are outlined in Table 3. The analysis demonstrates that treatment with TDM1 imposes a higher cost on the healthcare system compared to the trastuzumab arm.

**Table 3.** Budget Impact Analysis Results

Years	Budget Impact ($)
**1**	195,762
**2**	578,804
**3**	1,120,546

## 5. Discussion

In this study, we conducted a cost-utility and budget impact analysis of TDM1 in the adjuvant treatment of residual invasive HER-2-positive breast cancer. The findings indicate that the administration of TDM1 is cost-effective, as it provides additional QALYs at a cost below Iran's pharmacoeconomic threshold. One-way sensitivity analysis revealed that the model is sensitive to the probability of IDFS, the price of TDM1 and trastuzumab, and the discount rate of utility values and costs. However, in the majority of probabilistic sensitivity analysis simulations, the calculated ICER remained below the pharmacoeconomic threshold, with some simulations suggesting that TDM1 could impose lower costs compared to trastuzumab while improving QALYs.

Despite the cost-effectiveness analysis showing that TDM1 is cost-effective, the budget impact analysis demonstrated that treatment with TDM1 is associated with increased healthcare costs. This discrepancy arises from the differing time horizons of the analyses. The budget impact analysis considered only the first three years of TDM1 use, whereas the cost-effectiveness analysis adopted a lifetime horizon, capturing the long-term effects of TDM1 on costs. While TDM1 is more expensive and entails higher costs for managing adverse events, it ultimately reduces overall costs by prolonging OS, PFS, and distant recurrence-free survival.

Other studies have similarly demonstrated the cost-effectiveness of TDM1 for the treatment of residual invasive HER-2+ breast cancer. Evaluating the cost-effectiveness of TDM1 in Canada, Younis et al. designed a hypothetical Markov model for patients with HER-2-positive breast cancer with a lifetime horizon (21). They calculated a total incremental cost of 8,300$ for TDM1 versus trastuzumab from the healthcare system's perspective, with an improvement of 2.19 QALYs. The calculated ICER had a 97.5% likelihood of being cost-effective (20). Similarly, the analytical model by Magalhaes Filho et al., which employed a 30-year time horizon, determined a the quality-adjusted time with symptoms or toxicity and without symptoms or toxicity (Q-TWiST) gain of 3,812 years in quality-adjusted time without symptoms or toxicity for the TDM1 arm, with an ICER of 11,467.65$ in the United States and 3,332.73$ in Brazil, indicating cost-effectiveness in both countries (34). 

Guan et al. conducted a cost-utility analysis and found the ICER to be 1 - 2 times GDP per capita, below China's cost-effectiveness threshold (35). Goertz et al. carried out similar research on patients with HER-2-positive breast cancer with residual malignant tissue, concluding that TDM1 was the dominant option, providing greater efficacy at a lower cost (36).

Recently updated guidelines recommend TDM1 for patients with residual disease after neoadjuvant HER-2-directed therapy, as it improves IDFS and reduces the risk of distant recurrence (37, 38). Given that TDM1 has been shown to be cost-effective in Iran, it is essential for policymakers to make this treatment option available. Additionally, TDM1 serves as a second-line treatment for patients with HER-2+ breast cancer, but its cost-effectiveness in this context also requires evaluation in Iran.

Compared to patients with early-stage disease, individuals with metastatic breast cancer face significantly higher expenses. Expenditures increase steeply during the EOL phase, with EOL care in the last six months of life imposing a substantial economic burden (31). To provide a more accurate cost estimation, we included EOL costs in the calculation of the MP state costs. This study demonstrated that fewer patients receiving TDM1 experience metastatic progression compared to those receiving trastuzumab. Consequently, TDM1 reduces the likelihood of transitioning to the MP state and EOL, leading to lower MP state costs in the TDM1 group.

This study has several notable advantages. To comprehensively capture the outcomes of TDM1 therapy, we utilized a lifetime horizon for calculating the ICER. For greater accuracy in cost estimations, we calculated metastatic and EOL costs separately within the MP state. Additionally, we incorporated dynamic transition probabilities for each cycle. Importantly, since patients in any health state could die due to causes unrelated to the disease, we included this probability of death in our calculations.

However, this study has certain limitations. As no studies have specifically evaluated the impact of TDM1 on patients in Iran, we relied on input parameters derived from published literature. Similarly, EOL costs were calculated using data from prior studies, which may limit the precision of cost estimations tailored to the Iranian healthcare context.

### 5.1. Conclusions

In conclusion, the findings of this economic assessment research indicate that TDM1 is a cost-effective intervention compared to trastuzumab for patients with residual invasive HER-2-positive breast cancer, with a cost per QALY of 886$. However, the inclusion of TDM1 at the assumed price point is expected to increase the healthcare system's costs in terms of budget impact.

## Data Availability

The dataset presented in the study is available on request from the corresponding author during submission or after its publication. The data are not publicly available due to the need for providing additional explanations regarding the data sets.
